# A double-walled sapphire single-crystal gas-pressure cell (type III) for *in situ* neutron diffraction

**DOI:** 10.1107/S1600576721012048

**Published:** 2022-02-01

**Authors:** Raphael Finger, Thomas C. Hansen, Holger Kohlmann

**Affiliations:** a Inorganic Chemistry, Leipzig University, Johanisallee 29, Leipzig, 04103, Germany; b Institut Laue–Langevin, 71 avenue des Martyrs, Grenoble, 38000, France

**Keywords:** neutron diffraction, hydrogenation, neutron instrumentation, solid–gas, metal hydrides, sapphire, powder diffraction

## Abstract

*In situ* neutron diffraction is an important characterization technique for the investigation of many functional materials, *e.g.* for hydrogen uptake and release in hydrogen storage materials. A new sapphire single-crystal gas-pressure cell for elastic neutron scattering has been developed and evaluated; it allows conditions of 298 K and 9.5 MPa hydrogen pressure and 1110 K at ambient pressure. The pressure vessel consists of a sapphire single-crystal tube of 35 mm radius and a sapphire single-crystal crucible as sample holder. Heating is realized by two 100 W diode lasers. It is optimized for the D20 diffractometer, ILL, Grenoble, France, and requires the use of a radial oscillating collimator. Its advantages over earlier sapphire single-crystal gas-pressure cells are higher maximum temperatures and lower background at low and high diffraction angles. The deuterium uptake in palladium was followed *in situ* for validation, proving the potential of the type-III gas-pressure cell for *in situ* neutron diffraction on solid–gas reactions.

## Abstract 2

A new sapphire single-crystal gas-pressure cell for elastic neutron scattering to study solid–gas reactions is presented and evaluated; it allows conditions of 298 K and 9.5 MPa hydrogen pressure and 1110 K at ambient pressure.

## 1. Introduction

*In situ* X-ray and neutron diffraction have become very popular recently, because many interesting phenomena may be studied, such as phase transitions, chemical reactions, the wear of functional materials, the functioning of heterogeneous catalysts and battery materials, and industrial processes (Isnard, 2007 ▸; Pienack & Bensch, 2011 ▸; Hansen & Kohlmann, 2014 ▸; Yang *et al.*, 2016 ▸; Peterson *et al.*, 2017 ▸; Kohlmann, 2019 ▸). Time-resolved *in situ* neutron diffraction investigations are particularly interesting for materials in energy storage because processes of charging and discharging may be studied on an atomic level, *e.g.* in battery materials or in metal hydrides for hydrogen storage. Many phase transitions occur within the latter class of compounds, *e.g.* temperature- and pressure-driven structural and magnetic phase transitions (Kohlmann *et al.*, 2003 ▸; Goncharenko *et al.*, 1996 ▸). Hydrogen uptake and release from metal hydrides are of importance in view of the use as hydrogen stores (Møller *et al.*, 2014 ▸; Hansen & Kohlmann, 2014 ▸; Götze *et al.*, 2018 ▸).

For the *in situ* investigation of hydrogenation reactions under hydrogen (deuterium) gas pressure and at elevated temperature, specialized gas-pressure cells with laser heating have been developed and used in many studies (Peterson *et al.*, 2017 ▸; Götze *et al.*, 2018 ▸; Finger, Kurtzemann *et al.*, 2021 ▸; Finger, Hansen & Kohlmann, 2021 ▸). A sample environment based on single-crystal holders made of α-Al_2_O_3_ [called sapphire herein; see Finger, Kurtzemann *et al.* (2021 ▸)] allows for extremely low background without parasitic reflections, given proper orientation of the single crystal. Typically, they operate with laser heating at moderate temperatures and allow for a time resolution of less than 1 min (on high-intensity diffractometers). They can withstand high hydrogen (deuterium) gas pressures [*e.g.* 16.0 MPa at 298 K and 8.0 MPa at 655 K for type I (Finger, Kurtzemann *et al.*, 2021 ▸)], but are not designed for high temperatures. The highest temperature reached so far, 718 K, was recorded at ambient pressure with a type-II cell (Finger, Hansen & Kohlmann, 2021 ▸). In the present publication we describe a new type of sapphire single-crystal gas-pressure cell (type III). It allows for *in situ* neutron powder diffraction at considerably higher temperatures, thus enabling a wider range of solid–gas reactions to be studied. It is optimized for the D20 diffractometer (Hansen *et al.*, 2008 ▸), ILL, Grenoble, France, in high-resolution mode, with a take-off angle of 120° and a wavelength of about 187 pm, and requires the use of a radial oscillating collimator.

## 2. Concept, pressure vessel and sample holder of the type-III gas-pressure cell

**Figure 1:**
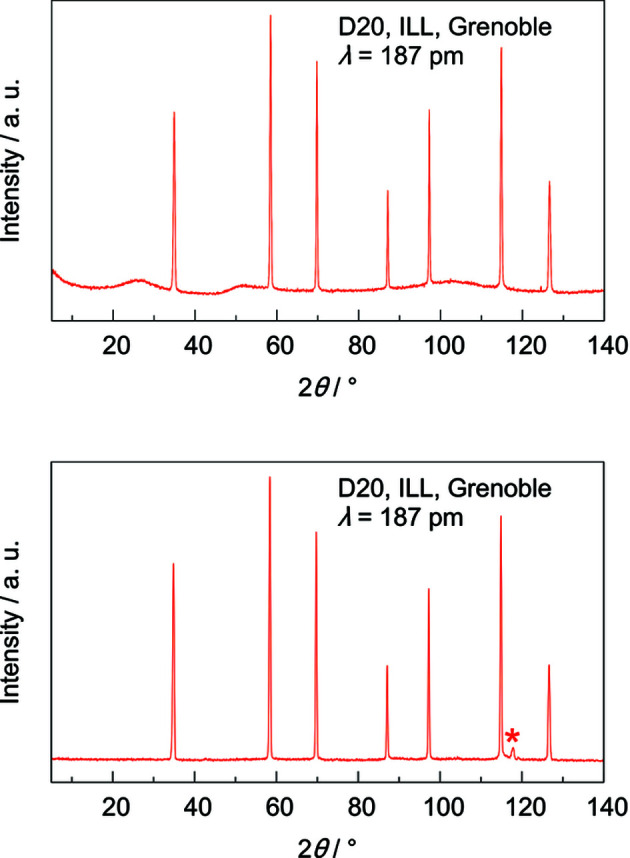
Neutron powder diffraction data of silicon powder in a quartz glass tube with an inner diameter of 4 mm and a wall thickness of 0.25 mm (5 ≤ 2θ ≤ 140°, normalized to have the same maximum intensity as the strongest reflection, 5 min measurement time; NUMOR 975353; https://doi.org/10.5291/ILL-DATA.5-24-605) (top) and in a type-III gas-pressure cell with a sapphire single-crystal crucible as sample holder with an inner diameter of 6 mm and a wall thickness of 1 mm (parasitic reflection from the sample environment marked with *, 5 ≤ 2θ ≤ 140°, 10 min measurement time; NUMOR 178819; https://doi.org/10.5291/ILL-DATA.5-24-639) (bottom). (NUMOR is the internal raw data labelling of the ILL, Grenoble, France.)

**Figure 2:**
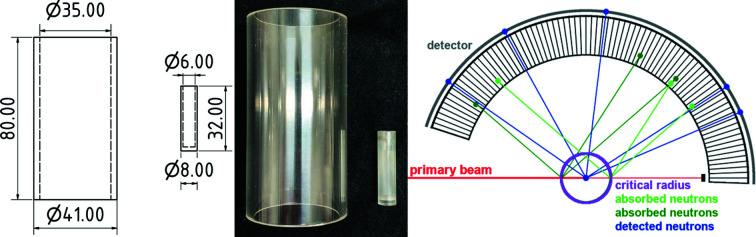
Technical schemes (left, length specification in mm) and photograph (middle) of the sapphire single-crystal pressure vessel and crucible; scheme of the radial oscillating collimator used at D20, ILL, Grenoble, France (right).

The previously presented gas-pressure cells of types I and II combine pressure vessel and sample holder in one work piece, a 10 cm-long machined sapphire single crystal with a 6 mm borehole (Finger, Kurtzemann *et al.*, 2021 ▸; Finger, Hansen & Kohlmann, 2021 ▸). In type-I gas-pressure cells the sample holder is fixed between a base mount and a flange joint with no connection in between, thus providing free optical access in the diffraction plane (Finger, Kurtzemann *et al.*, 2021 ▸). The type-II gas-pressure cell uses a single corpus around the sample holder with windows for primary and scattered neutron beams, optical surveillance and laser heating (Finger, Hansen & Kohlmann, 2021 ▸). For the cell presented herein, type III, the tasks of holding pressure and holding a sample are uncoupled, *i.e.* the pressure vessel and sample holder are two different work pieces. This results in more degrees of freedom for physical dimensions and materials, and offers the opportunity to optimize both pieces for their individual task. For example, the sample holder may have a smaller wall thickness, since it does not need to withstand the gas pressure, reducing the contribution of the sample holder and thus resulting in a higher quality of the diffraction data. Furthermore, a smaller wall thickness for the sample holder means that a much wider variety of materials can be used as sample holder, *e.g.* parasitic reflections from polycrystalline materials or the structured background of amorphous materials may be small or negligible. Such materials were ruled out of consideration as a pressure vessel by Finger *et al.* but meet the requirements to be potential sample holders for the type-III gas-pressure cell. Therefore, we firstly focused on quartz glass (Hilgenberg GmbH, Malsfeld, Germany) as it is available in small wall thicknesses of less than 1 mm, even with an inner diameter of 4 mm. Though quartz glass showed a highly structured background despite its small wall thickness of 0.25 mm (Fig. 1 ▸, top), a sapphire single-crystal crucible, 32 mm in height, 6 mm inner diameter with a wall thickness of 1 mm, has been chosen as sample holder (Fig. 2 ▸). Despite the much higher wall thickness, the crucible offers a better overall data quality because of its low background contribution for proper orientation of the single-crystal crucible. For the pressure vessel, a sapphire single-crystal tube is used (Impex HighTech GmbH, Münster, Germany). The material is a common choice for *in situ* diffraction experiments (Finger, Kurtzemann *et al.*, 2021 ▸; Götze *et al.*, 2018 ▸). Neutron activation, neutron absorption for thermal neutrons and incoherent scattering are low for sapphire (Sears, 1992 ▸). Furthermore, potential irradiation damage occurs only at neutron doses as high as 10^18^ neutrons cm^−2^ (Abdukadyrova, 2005 ▸), which is way above the expected value for a typical neutron diffraction experiment considering the neutron flux at D20 [10^7^ neutrons s^−1^ cm^−2^ for λ = 1.87 Å, Ge(115) monochromator, 120° take-off angle (Hansen *et al.*, 2008 ▸)].

To completely avoid the contribution of the pressure vessel to the resulting diffraction pattern, a minimum diameter of 35 mm is dictated by the radial oscillating collimator used at D20 (Fig. 2 ▸). Equation (1) of Finger, Kurtzemann *et al.* (2021 ▸) gives a maximum pressure of 63 MPa at 298 K for a wall thickness of 3 mm and about 43 MPa at 770 K. Including a safety factor of four to account for mechanical stress and potential imperfections of the single crystal, the use of the type-III gas-pressure cell is limited to a maximum overpressure of 10 MPa. So far, a maximum pressure of 9.5 MPa at room temperature has been tested. Higher pressures may be realized but have not yet been tested, because they are not necessary for the kind of *in situ* experiment that we have so far carried out, typically with 0.1 < *p* < 10 MPa and 300 < *T* < 700 K.

## 3. Double-walled sapphire single-crystal gas-pressure cell (type III)

**Figure 3:**
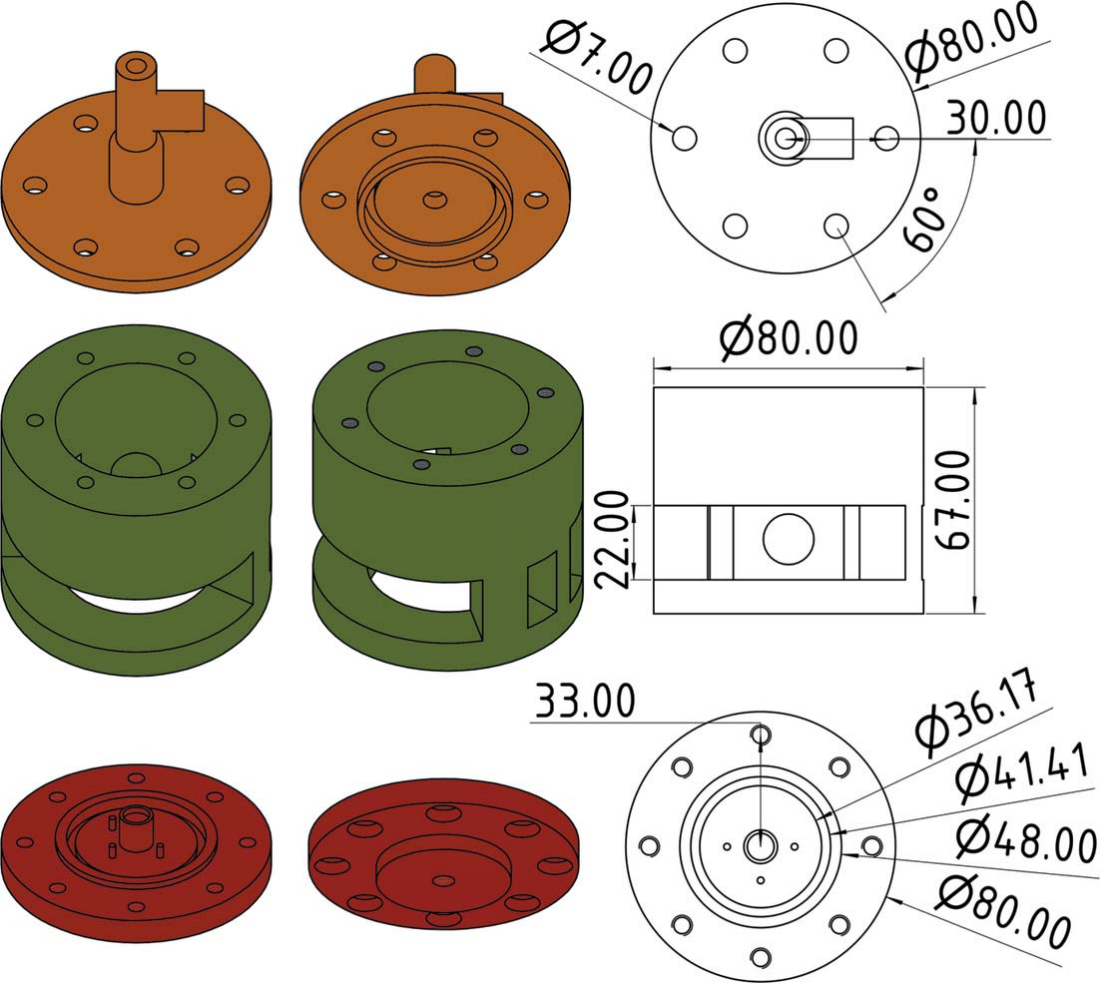
Main parts of the type-III gas-pressure cell: top plate (top), corpus (middle), bottom plate (bottom); length specification in mm.

The gas-pressure cell has three main parts: a bottom plate, a corpus and a top plate. All main parts are made of austenitic chromium–nickel stainless steel (EN 1.4301/AISI 304). The bottom plate has a diameter of 80 mm and a thickness of 8 mm. The corpus is connected to it via eight M6 Allen screws. To fix the cell on the diffractometer, the bottom plate has an ISO metric M8 thread (Fig. 2 ▸). The grooves in the plate, 36.17 and 41.41 mm, are for the NBR 70 O-ring seal (Nitrile Butadiene Rubber 70 Shore, usable up to 393 K) to realize gas pressure. The outer rim keeps the O-ring in position. The corpus is the main part of the gas-pressure cell as it connects the bottom and top plates. It is painted with neutron-absorbing gadolinium oxide varnish to prevent neutron activation. The top plate is similar to the bottom plate with six boreholes to screw it to the corpus. A T-piece is welded on top of the plate for gas feed and temperature measurement. A thermocouple covered with neutron-absorbing gadolinium oxide varnish may be screwed into a 6 mm Swagelok nut in order to measure temperature in the reaction chamber (Figs. 3 ▸ and 4 ▸). Temperature surveillance with a pyrometer as for types I and II did not work, because only the temperature of the pressure vessel was measured. However, the opening that was originally designed for the pyrometer can be used for optical surveillance, which is valuable for reactions including colour changes.

The higher gas volume, compared with the type-I and -II cells, is compensated with an inlet, lowering the gas volume from 67.3 to 27.3 cm^3^ and thus minimizing risk. The inlet is 32 mm in diameter and 60 mm in height and has a 16 mm opening on the bottom for the sample holder and its rest. So as not to interfere with the primary beam or scattered neutrons, the inlet has the same openings as the corpus (Figs. 3 ▸ and 4 ▸). To prevent neutron activation, the corpus and inlet are painted with a neutron-absorbing varnish as used for the type-II gas-pressure cells (Finger, Hansen & Kohlmann, 2021 ▸). The inlet is fixed on the bottom plate via three pins, allowing a reproducible positioning. The central rest for the sample holder is not higher than the painted inlet, thus preventing neutron activation.

## 4. Assembly

As a first step, an O-ring seal (Fig. 5 ▸, black) is placed onto the bottom plate (Fig. 5 ▸, red). Then, the corpus (Fig. 5 ▸, green) is screwed to the bottom plate. The crucible is placed on the sample stage in the middle of the bottom plate and the inlet (Fig. 5 ▸, blue) is carefully put on top, being clicked into the three pins (Fig. 3 ▸, bottom left) to allow a reproducible positioning. Afterwards, the sapphire tube as pressure vessel (Fig. 5 ▸, grey) is placed on the O-ring. The threaded bars (ISO metric M6, 40 mm long) are turned all the way in the corresponding threaded boreholes on the top of the corpus. The upper seal is positioned on the top of the sapphire tube and the top plate is put carefully on top. Finally, the gas-pressure cell is screwed together by fastening the nuts on the threaded bars finger tight and then turning them crosswise step by step up to a torque of 1.2 N m. The thermocouple and gas supply may be attached afterwards. To realize the gas-tight sealing of the thermocouple with an inner diameter of 6 mm, a corresponding 6 mm Swagelok screw connection is attached and fixed.

## 5. Tested temperature and pressure conditions

In the course of our *in situ* studies, typical temperature–pressure conditions for hydrogenation reactions were applied, which are considerably lower than the maximum values given above. As breaking tests were not performed, we cannot state the maximum temperature–pressure conditions, but only those tested and found to be safe (Table 1 ▸ and Appendix *A* [App appa]). The maximum temperature–pressure conditions might be higher. The temperatures are much higher than for type-I and -II cells (Finger, Kurtzemann *et al.*, 2021 ▸; Finger, Hansen & Kohlmann, 2021 ▸), which is clearly one of the advantages of decoupling the pressure vessel and sample holder, albeit at the cost of reduced maximum pressure. For technical reasons, high temperatures have so far only been tested in air, but are expected to hold also for hydrogen pressures.

For the exact sample temperature determination on a neutron diffractometer, calibration was performed via the known thermal expansion of the palladium lattice (Dutta & Dayal, 1963 ▸) by a procedure described previously (Finger, Kurtzemann *et al.*, 2021 ▸).

## 6. Validation of the double-walled sapphire single-crystal gas-pressure cell

An ω scan (data collection while turning around the principal goniometer axis) of the type-III gas-pressure cell (Fig. 6 ▸, left) shows a section of about 5° with no significant change in absolute intensities (Fig. 6 ▸, middle) and of about 8° with unaffected reflection/background ratio (Fig. 6 ▸, right). Typically, less than 5° is needed for the fine tuning of the ω position, according to our experience with the single crystals used in type-I and -II cells (Finger, Kurtzemann *et al.*, 2021 ▸; Finger, Hansen & Kohlmann, 2021 ▸). Thus, the window is wide enough to allow for proper alignment of the type-III gas-pressure cells.

To validate the type-III gas-pressure cell, the hydrogenation of palladium was studied. It is well suited due to a great body of knowledge, the high symmetry of the starting material and the resulting palladium deuteride, and favourable neutron scattering lengths. For the reaction, deuterium was chosen instead of hydrogen because of its lower incoherent scattering cross section. Palladium powder (99.95%, mesh size < 150 µm, GoodFellow) was dried under vacuum (1 Pa) at 773 K for 24 h and handled under argon afterwards. In the *in situ* experiment, palladium powder was heated under vacuum (9 kPa) to 360 (2) K before injections of deuterium gas were slowly added. Rietveld refinements were performed using *FullProf* with pseudo-Voigt profile functions (Rodríguez-Carvajal, 1993 ▸). Starting models for palladium and the α and β phases of palladium deuteride were taken from the work of Kohlmann *et al.* (2013 ▸).

When hydrogen is absorbed by palladium, hydrogen atoms occupy octahedral voids; tetrahedral void occupation as seen above the critical point of the phase diagram (Pitt & Gray, 2003 ▸) was not observed, as we performed the experiment at temperatures and pressures below the critical point. The deuterium contents of palladium deuteride in this *in situ* experiment (Table 2 ▸) are in good accordance with the experimental data (Wicke & Blaurock, 1987 ▸) (Figs. 7 ▸, 8 ▸ and 9 ▸). The lattice parameter and isotropic displacement parameters (*B*_iso_) of the atoms are reasonable (Table 2 ▸).

## 7. Data quality of the type-I, -II and -III gas-pressure cells

**Figure 4:**
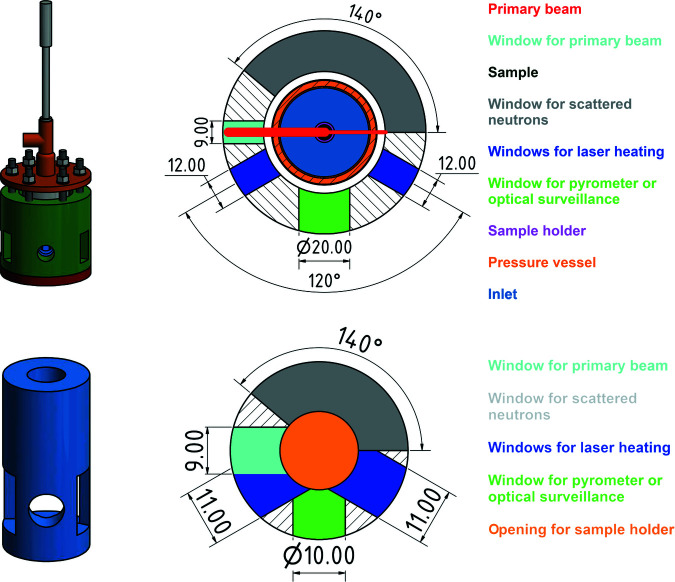
Type-III gas-pressure cell (top) and aluminium inlet (bottom) in three-dimensional representation (left), and section of the plane of diffraction (right); length specification in mm.

**Figure 5:**
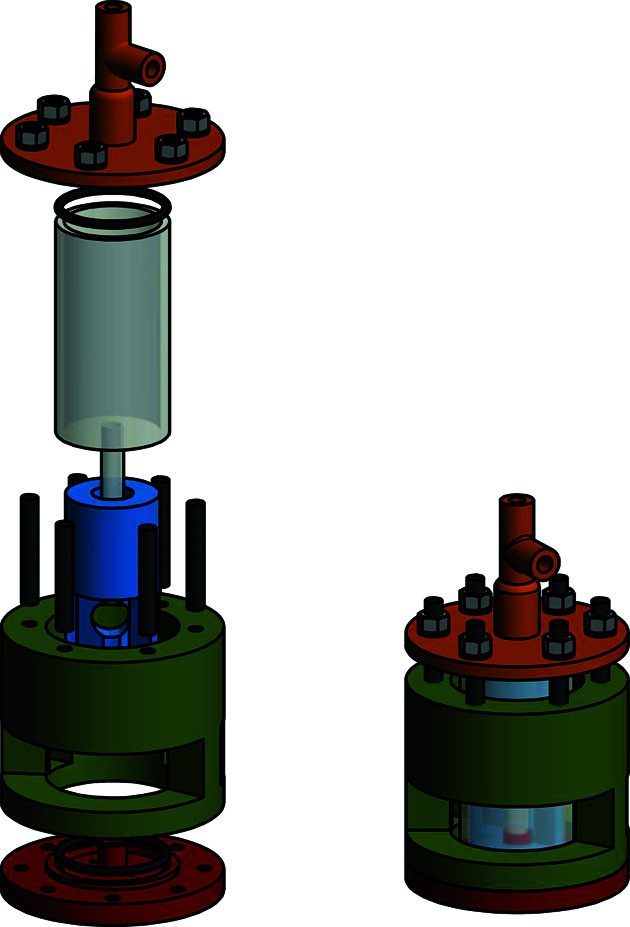
Exploded-view drawing (left) and three-dimensional representation (right) of the type-III gas-pressure cell.

**Figure 6:**
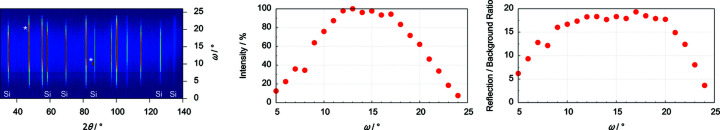
False-colour plot of an ω scan of silicon powder in an aluminium crucible performed on D20 at ILL in 1° steps of ω and with 60 s data accumulation per ω position. Highest intensity in red, lowest intensity in blue, parasitic reflection from the sample environment marked with * (left). Relative intensities of the silicon 111 reflection as a function of ω (middle) and reflection/background ratio as a function of ω (right). (NUMOR 138794; http://doi.org/10.5291/ILL-DATA.5-24-621).

**Figure 7:**
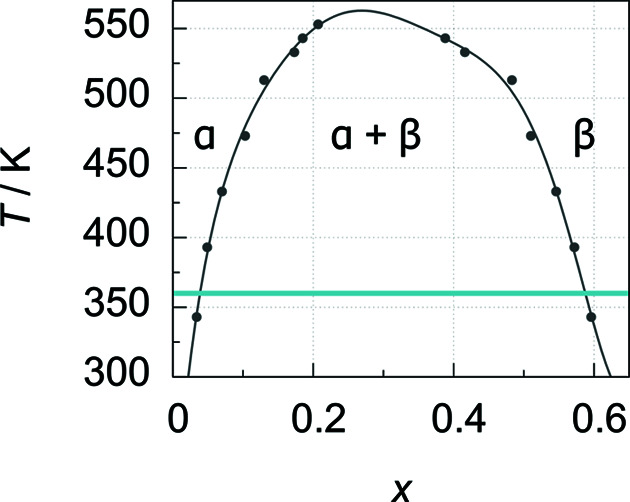
Palladium–deuterium phase diagram (temperature versus deuterium content *x* in PdD*_x_*); based on absorption isotherms [data from Wicke & Blaurock (1987 ▸); grey points] fitted with a polynomial function (grey line) and the temperature used for validation of the gas-pressure cell (blue line).

**Figure 10:**
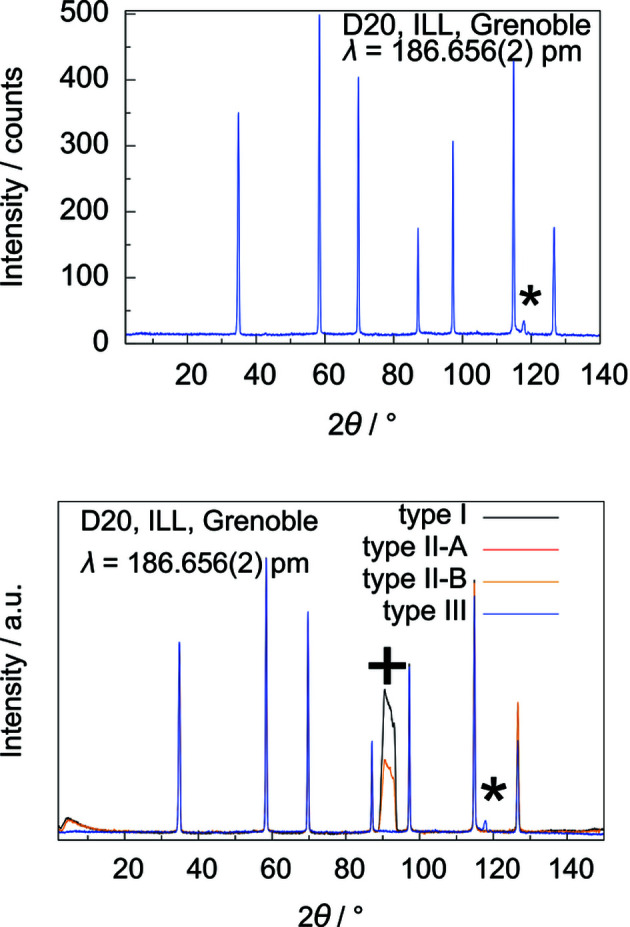
Neutron diffraction data of the type-III gas-pressure cell with silicon powder (2 ≤ 2θ ≤ 140°, parasitic reflection marked with *, 10 min measurement time; NUMOR 178819) (top), and comparison of the normalized neutron diffraction data for type-I, -II and -III gas-pressure cells (sample holder contribution from type-I and -II sample holders marked with +, parasitic reflection of the type-III sample environment marked with *; NUMORs 178776, 178781, 178810, 178819) (bottom) (https://doi.org/10.5291/ILL-DATA.5-24-639).

To evaluate the overall data quality, the background at low and high 2θ values and the reflection/background ratios are considered (Table 3 ▸ and Fig. 10 ▸, top). The same amount of sample has been used for measurements with the type-I and -II gas-pressure cells, and only the measurement time is different (10 min for type III instead of 20 min for types I and II); thus the measurements are comparable for normalized representations (Fig. 10 ▸, bottom). Details for the type-I and -II measurements are provided by Finger, Hansen & Kohlmann (2021 ▸). The striking feature of the type-III compared with the type-I and -II gas-pressure cells is a very low background at low and high angles [calculated as by Finger, Hansen & Kohlmann (2021 ▸), standard deviation of intensities considered]. The air and deuterium gas scattering, visible at low 2θ angles, is very small, as a result of the inlet (Figs. 4 ▸ and 5 ▸).

**Figure 11:**
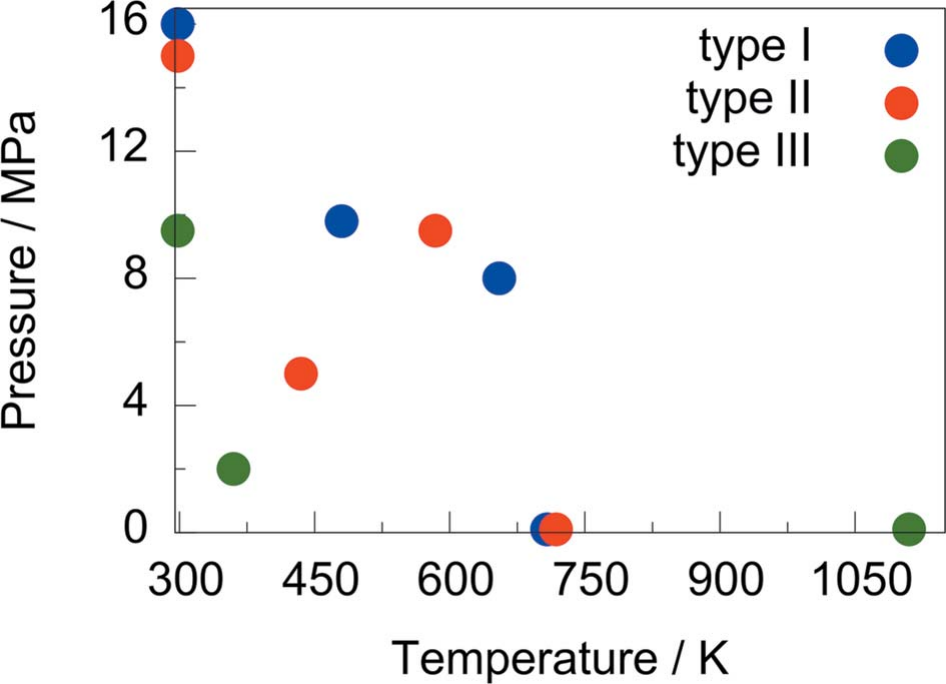
Comparison of tested temperature and pressure conditions with type-I (Finger, Kurtzemann *et al.*, 2021 ▸), type-II (Finger, Hansen & Kohlmann, 2021 ▸) and type-III (this work) gas-pressure cells.

The tested temperature and pressure conditions of all three types of gas-pressure cells are compared in Fig. 11 ▸. The type-I cell has been used for over ten years and thus has well known operating conditions. Type-II and -III cells are mainly used for laboratory tests and are less well studied under real conditions. The type-III cell offers most potential at higher temperatures with a maximum of about 1110 K reached at 0.1 MPa air.

## 8. Conclusions

In this work we present a new design for a sapphire single-crystal gas-pressure cell for *in situ* neutron diffraction. It is suitable for hydrogenation reactions up to 10 MPa and reached a maximum temperature of about 1110 K. Its advantage over earlier designs is a lower diffraction background at low and high diffraction angles. The decoupling of the sample holder and pressure vessel allows for an optimization of both parts independently. Moreover, the new design allows for higher sample temperatures and thus enables further studies of thermal stability, decomposition, and reversibility of hydrogen uptake and release. The adaptation to gases other than hydrogen should be straightforward and will allow a wide range of *in situ* experiments on solid–gas reactions in the future.

## Supplementary Material

Raw data: NUMOR 975353: https://doi.org10.5291/ILL-DATA.5-24-605

Raw data: NUMOR 178819: https://doi.org/10.5291/ILL-DATA.5-24-639

Raw data: NUMOR 138794: http://doi.org/10.5291/ILL-DATA.5-24-621

Raw data: NUMORs 145703, 145704, 145748: https://doi.org/10.5291/ILL-DATA.5-22-767

Raw data: NUMORs 178819, 178776, 178781, 178810, 178819: https://doi.org/10.5291/ILL-DATA.5-24-639

## Figures and Tables

**Figure 8:**
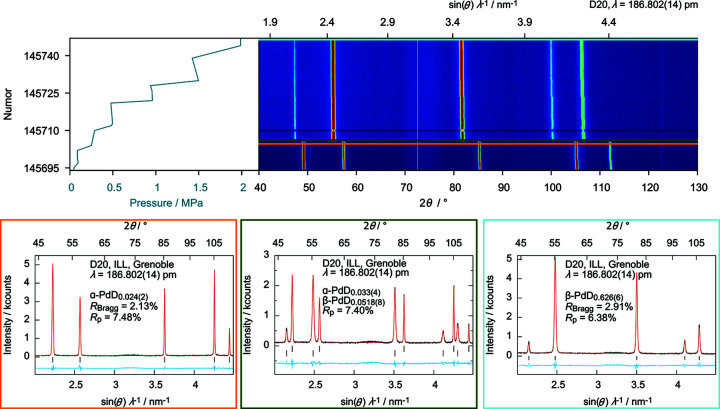
*In situ* neutron powder diffraction of the reaction of palladium powder with deuterium at 360 (2) K. Top: pressure profile (left), diffraction data as false-colour plot with highest intensity in red and lowest intensity in blue (right). Bottom: Rietveld refinements (observed intensities, *I*_obs_, in black, calculated intensities, *I*_calc_, in red, difference, *I*_obs_ − *I*_calc_, in blue, excluded region 72.6 ≤ 2θ ≤ 72.7° due to detector failure, 2 min per NUMOR; https://doi.org/10.5291/ILL-DATA.5-22-767) of the crystal structures of α-PdD_0.024 (2)_ (NUMOR 145703, left, corresponding to orange line top right), α-PdD_0.033 (4)_ and β-Pd_0.518 (8)_ (NUMOR 145704, middle, corresponding to dark-green line top right), and β-PdD_0.626 (6)_ (NUMOR 145748, right, corresponding to light-blue line top right).

**Figure 9:**
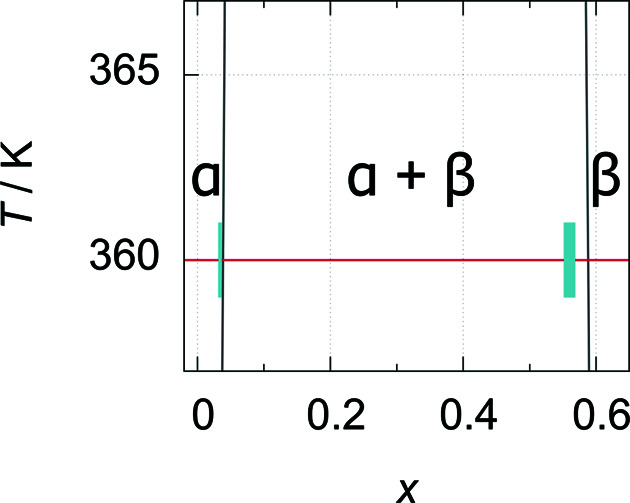
Part of the palladium–deuterium phase diagram (temperature versus deuterium content *x* in PdD*_x_*): blown-up section of Fig. 7 ▸ at 0.25 MPa deuterium gas pressure, data from Wicke & Blaurock (1987 ▸), experimentally determined deuterium content at 0.25 MPa and 360 (2) K (red line) for α-PdD_0.033 (4)_ and β-PdD_0.560 (5)_ with ± as blue boxes.

**Figure 12:**
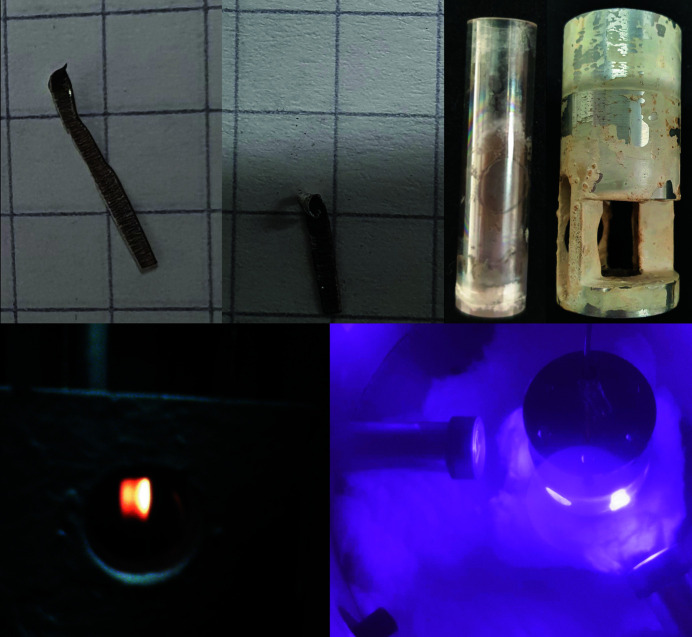
Photographs of the type-III gas-pressure cell and *in situ* setup during temperature tests: the piece of aluminium before and after experiment C (see Appendix *A* [App appa]) (top left), crucible with the aluminium melted into the silicon and inlet after experiment C (top right), webcam view through an infrared absorbing glass filter along the pyrometer window at 1110 K and a laser power of 91.5 W during experiment A (bottom left), and the *in situ* setup at 803 K and a laser power of 62.6 W without filters taken with a mobile phone (bottom right).

**Table 1:** Maximum gas-pressure (*p*) and temperature (*T*) conditions tested for the type-III sapphire single-crystal gas-pressure cell with a sapphire tube of 35 mm inner diameter and 3 mm wall thickness as pressure vessel

Gas	*p* (MPa)	*T* (K)
Air	0.1	1110
D_2_	2.0	360
H_2_	9.5	298

**Table 2:** Crystal structures of palladium deuterides PdD*_x_* at 360 (2) K and between 0.085 and 2.0 MPa D_2_ pressure (space-group type *Fm* 3 *m*, Pd in 4*a* 0 0 0, D in 4*c* ½ ½ ½)

Phase	*p*(D_2_) (MPa)	Lattice parameter (Å)	Deuterium content *x*	*B*_iso_(Pd) (Å^2^)	*B*_iso_(D) (Å^2^)
α-PdD_*x*_	0.085	3.90411 (3)	0.024 (2)	1.18 (4)	4†
α-PdD_*x*_	0.25	3.90681 (4)	0.033 (4)	1.41 (5)	4†
β-PdD*_x_*	0.25	4.03749 (15)	0.560 (5)	1.39 (7)	3.85 (14)
β-PdD*_x_*	2.0	4.04788 (9)	0.626 (6)	1.35 (6)	4.09 (9)

† Fixed as no proper refinement was possible due to the small amount of deuterium, value taken from type-II validation (Finger, Hansen & Kohlmann, 2021 ▸).

**Table 3:** Reflection/background ratio *R*_I_ of all silicon reflections measured from neutron powder diffraction data using the type-III gas-pressure cell

*hkl*	2θ (°)	*R*_I_
111	34.85	25.8 (3)
220	58.35	33.0 (4)
311	69.75	29.7 (4)
400	87.10	11.3 (2)
331	97.25	19.4 (3)
422	114.90	27.1 (3)
511/333	126.65	13.6 (3)

## Appendix A Validation of temperature measurements

In order to confirm the measured temperatures in the type-III gas-pressure cell, three different experiments were performed using the same crucible with a 20–22 mm-high filling of silicon powder and heating by two diode lasers running at 91.5 W. A thin thermocouple was put into the crucible, measuring the temperature at the position of maximum laser heating to be 1110 K (experiment A). The temperature of the top surface of the sample was measured by a pyrometer to be 1013 K (experiment B). A long thin piece of aluminium was stuck into the silicon powder, reaching from the position of maximum laser heating to the air above the heating zone, while a thermocouple at 3 mm depth measured 839 K (experiment C). About half of the aluminium melted, which is in accordance with the temperature gradient and proves that the maximum temperature was at least as high as the melting point of aluminium (933 K). Additionally, the gadolinium oxide varnish showed signs of thermal decomposition, which is another indication of high sample temperatures (Fig. 12 ▸).
